# Feasibility of Wave Intensity Analysis in Patients With Conotruncal Anomalies Before and After Pregnancy: New Physiological Insights?

**DOI:** 10.3389/fped.2020.557407

**Published:** 2021-03-04

**Authors:** Maria Victoria Ordonez, Sandra Neumann, Massimo Caputo, Stephanie Curtis, Giovanni Biglino

**Affiliations:** ^1^Bristol Heart Institute, University Hospitals Bristol, Bristol, United Kingdom; ^2^Bristol Medical School, University of Bristol, Bristol, United Kingdom; ^3^National Heart and Lung Institute, Imperial College London, London, United Kingdom

**Keywords:** conotruncal anomalies, pregnancy, aorta distensibility, aorta diameter, wave intensity analysis

## Abstract

**Background:** Conotruncal anomalies (CTA) are associated with ongoing dilation of the aortic root, as well as increased aortic stiffness, which may relate to intrinsic properties of the aorta. Pregnancy hormones lead to hemodynamic changes and remodeling of the *tunica media*, resulting in the opposite effect, i.e., increasing distensibility. These changes normalize post-pregnancy in healthy women but have not been fully investigated in CTA patients.

**Methods:** We examined aortic distensibility and ventriculo-arterial coupling before and after pregnancy using cardiovascular magnetic resonance (CMR)-derived wave intensity analysis (WIA). Pre- and post-pregnancy CMR data were retrospectively analyzed. Aortic diameters were measured before, during, and after pregnancy by cardiac ultrasound and before and after pregnancy by CMR. Phase contrast MR flow sequences were used for calculating wave speed (*c*) and intensity (WI). A matched analysis was performed comparing results before and after pregnancy.

**Results:** Thirteen women (*n* = 5, transposition of the great arteries; *n* = 6, tetralogy of Fallot; *n* = 1, double outlet right ventricle, *n* = 1, *truncus arteriosus*) had 19 pregnancies. Median time between delivery and second CMR was 2.3 years (range: 1–6 years). The aortic diameter increased significantly after pregnancy in nine (*n* = 9) patients by a median of 4 ± 2.3 mm (range: 2–7.0 mm, *p* = 0.01). There was no difference in c pre-/post-pregnancy (*p* = 0.73), suggesting that increased compliance, typically observed during pregnancy, does not persist long term. A significant inverse relationship was observed between *c* and heart rate (HR) after pregnancy (*p* = 0.01, *r* = 0.73). There was no significant difference in cardiac output, aortic/pulmonary regurgitation, or WI peaks pre-/post-pregnancy.

**Conclusions:** WIA is feasible in this population and could provide physiological insights in larger cohorts. Aortic distensibility and wave intensity did not change before and after pregnancy in CTA patients, despite an increase in diameter, suggesting that pregnancy did not adversely affect coupling in the long-term.

## Introduction

Conotruncal cardiac anomalies (CTA) include a variety of congenital heart defects, such as tetralogy of Fallot (ToF), *truncus arteriosus* (TA), double outlet right ventricle (DORV), and transposition of the great arteries (TGA). These defects represent 5–10% of congenital heart disease (1) and generally lead to severe cyanosis, necessitating repair in the newborn period or early in infancy. A common observation in CTA is thoracic aortic dilation. Landmark work by Niwa demonstrates that the incidence of aorta dilation (AD) in adults with repaired ToF approaches 15% despite early ToF repair (1). Progressive dilation of the neo-aortic root is out of proportion to somatic growth in TGA after repair with arterial switch surgery (2), and AD is also found in the majority of patients with TA who survive initial repair (3).

Some independent variables can have an impact on the structure and size of the aorta, such as age, hypertension, smoking, diabetes, and pregnancy. However, there are a few hypotheses for why AD in CTA occurs principally. The first is that AD occurs due to hemodynamic stress on the aorta from an early right-left shunt. Evidence to support this hypothesis includes data showing that AD is worse with worsened degrees of right ventricular outflow tract stenosis and in patients with pulmonary atresia more than in patients with pulmonary stenosis (4). A second hypothesis is that volume loading of the aorta, *via* a surgical systemic-to-pulmonary shunt, will increase flow through the aortic valve, thus leading to dilation of the proximal aorta *via* increased wall stress (5). A longer duration between shunting and complete repair has been found to correlate with AD in repaired ToF (3–5). Other observations that have been associated with larger aortic dimensions in ToF include having a right rather than left aortic arch and male sex (5, 6). Recent evidence demonstrates that early ToF repair does not normalize aortic compliance and that abnormal ascending aortic flow patterns based on 4D flow cardiac magnetic resonance (CMR) are prevalent and might affect long-term cardiac performance (7).

Pivotal questions are whether these abnormalities are inherent or acquired, whether congenital heart disease plays a causal or facilitating role and whether genetic determinants are important. Recent data suggest that CTA may be associated with a primary problem with aortic histology and should be considered a true aortopathy (6). Patients with CTA present with impaired distensibility of the aorta (6). Interestingly, patients with ToF present with a higher degree of aortic dilation and aorta distensibility than d-TGA patients (8). In addition, in patients with d-TGA, impaired distensibility has only been found at the level of the ascending aorta, whereas in ToF patients both the ascending and descending aorta have been shown to be affected (9).

In this context, it is known that pregnancy represents an independent risk factor as it impacts the elastic properties of the aorta (9). This is due to a combination of the hemodynamic stress of pregnancy in producing changes in arterial structure and integrity and increasing the risk of arterial dissection and rupture, as well as changes to the independent role of female hormones. These structural changes are believed to regress to pre-pregnancy levels after delivery; however, whether or not these changes persist is unknown (9, 10).

Aortic distensibility, as well as ventriculo-arterial coupling, can be assessed by an established mathematical technique called wave intensity analysis (WIA) (11). This is traditionally derived from pressure and velocity data, but it can also be formulated using velocity and area, thus rendering the analysis applicable to phase contrast cardiac magnetic resonance (PC-CMR) (12).

This study aims to evaluate possible changes in aortic distensibility and ventriculo-arterial coupling in a CTA cohort before and after pregnancy non-invasively, based on the hypothesis that stiffness of the aorta and mechanical ventricular properties would be adversely affected by the hemodynamic stress and hormonal changes imposed by pregnancy.

## Methods and Materials

This was a retrospective feasibility study performed at a single NHS site. Therefore, no approval from the NHS UK research and ethics committee was sought in line with the Health Regulatory Authority guidelines.

### Participants

The inclusion criteria were (i) conotruncal anomaly and (ii) phase contrast scan in the aorta available before and after pregnancy. Twenty-eight women with surgically repaired congenital conotruncal anomalies who had undergone pregnancy and had cardiac magnetic resonance scans at the University Hospitals Bristol NHS Foundation Trust were retrospectively assessed. Of these, 15 in total were removed from the analysis: 13 women had the appropriate scans available only at one time point (i.e., before or after pregnancy) and a further two patients who had insufficient scan quality (velocity encoding artifacts).

Thirteen patients (*n* = 13) were therefore entered into the analysis. Of these, five had transposition of the great arteries, six had tetralogy of Fallot, one had a double outlet right ventricle, and one had *truncus arteriosus*. Baseline characteristics are reported in Table 1.

**Table 1:** Baseline characteristics.

**CHD**	***n***	**Age at pregnancy** **(years)**	***N* pregnancies**	**Weight** **(kgs)**	**Height (cm)**	Body mass index (BMI)^*^	**HTA**	**Blood pressure** **Pre vs. Post (mmHg)**	**Smoking**
ToF	7	29.5	10	58.3	159.5		1	112/70	1
TA	1	19	1	53.0	156.0		0	108/70	0
d-TGA	5	26	8	55.0	161.0		0	119/65	3
**Total**	13	34	19	58.3	159.5	24.6	1	116/64 vs. 120/67	4

* *Weight, height, and BMI (body mass index) values correspond to 2.3 years post pregnancy. Data are expressed as means ± s.d*.

*HT, arterial hypertension; ToF, tetralogy of Fallot; TA, truncus arteriosus; d-TGA, transposition of the great arteries; CHD, congenital heart defect*.

### Image Acquisition

Clinical ECG-gated phase contrast MR angiography in the ascending aorta was acquired on a 1.5T Siemens Avanto Magnetom (Siemens Healthineers, Erlangen, Germany). Flow data were acquired in the axial plane above the valves during free breathing. The acquisition parameters of the phase contrast MR angiography (PC-MRA) were as follows: field of view = 250 mm, slice thickness = 5 mm, matrix = 256 × 100, voxel = 1.0 × 1.0 × 5 mm, TR = 10.95 ms, TE = 2.61 ms, phase reconstructions = 20–30, velocity encoding (VENC) = 150–500 cm/s. See Table 2.

**Table 2:** Imaging parameters, presented as median (range).

	**Before**	**After**	***P*-value**
VENC (cm/s)	200 (150–400)	200 (150–500)	0.84
TR (ms)	31 (29.9–47.15)	47.15 (29.9–47.15)	0.23
TE (ms)	3.8 (2.0–3.8)	2.0 (2.0–2.18)	0.025
Reconstructed phases	30 (20–30)	30 (20–30)	0.43

### Segmentation

The ascending aortas were manually segmented using the magnitude image (CVI42, Circle Cardiovascular Imaging Inc, Calgary, Canada). This allowed for the quantification of the average blood flow velocity (U) in each frame over the cross-sectional area (A).

### Aortic Dimensions

Echocardiographic data were reviewed in repaired CTA at baseline (1 year before pregnancy), during pregnancy in the third trimester, and >3 years after pregnancy. Two-dimensional echo measurements of the diameter of the sinus of Valsalva and sinotubular junction were made in the parasternal long-axis view at end-diastole using the inner edge-inner edge technique.

Aortic measurements from cross-sectional cardiac CMR images were taken before and after pregnancy, considering only changes >1.5 mm (pixel size).

### Physiological Parameters

Within the segmented area, cardiac output (L/min), mean flow velocity (cm/s), regurgitation fraction (%), and net negative volume of the flow (ml) were quantified. A 3-lead ECG measured the mean heart rate during the scan. Volumetric data were also calculated (end-diastolic volume EDV, end systolic volume ESV, and stroke volume SV). A ratio SV/ESV was derived from these as an accepted indicator of ventricular-vascular coupling (13).

### Wave Intensity Analysis

The A and U data were smoothed by the application of a Savitsky-Golay filter with a window size of 11 and a second-degree polynomial. Following the wave intensity analysis method outlined by Biglino et al. (12), the *U*-ln*A* loop was plotted and the linear portion of the slope selected during early systole to calculate wave speed (c) where *c* = *dU*/*d*ln*A*. The net wave intensity, *dI* = *dUd*ln*A*, as well as the forward and backward wave components were fitted in MATLAB (The MathWorks Inc., Natick, MA, USA). Wave energy was quantified by the area under the curve of the forward compression wave (FCW), forward expansion wave (FEW), and backward compression wave (BCW).

Wave intensity defined as *dI*_(A)_ = *dUd*ln*A* differs from the definition of wave intensity *dI*_(P)_ = *dPdU* by a difference in dimensions [wave intensity *dI*_(P)_ has the units *W*/m^2^ and *dI*_(A)_ has the units m/s] (14, 15). However, it can be shown that they differ only by a scaling factor, ρ*c*^2^, where ρ = blood density (16). We report *dI*_(A)_ as wave energy in m/s.

### Statistical Analysis

Normality of the data was assessed by D'Agostino-Pearson test of normality.

The FEW and BCW, as well as the regurgitation fraction and net negative volume, did not pass normality and was analyzed using non-parametric tests, namely Wilcoxon matched-pairs signed rank test. All other comparisons were run as Student paired *t*-tests, comparing the measure before and after pregnancy. Correlations were assessed by Pearson's correlation coefficient. All analyses were performed in GraphPad Prism version 7.0d (GraphPad, La Jolla, CA, USA). Data are reported as mean ± standard deviation. Alpha was set at 0.05. In the figures, the asterisk signifies the following: *p < 0.05, **p < 0.01, ***p < 0.001.

## Results

### Demographics

Thirteen pregnant women were analyzed, with a mean age at pregnancy of 28.5 ± 4.6 years. The total number of pregnancies was 19. Baseline data, including blood pressure, smoking status, body mass index, and age at pregnancy are reported in Table 3.

**Table 3:** Baseline data.

**CHD pathologies (*****n*****):**	
Arterial Switch	2
Atrial Switch	3
TA	1
TOF	7
**Corrective procedures (*n*)**	
TAP	1
TAP/PVR	1
DORV/PVR	1
TOF/PVR	3
TOF	1
Senning	3
AVR/PVR	1
Age at last pregnancy (years)	27.5 ± 4.7
Number of pregnancies	1.5 ± 0.9
Time from last pregnancy to MRI (years)	3.2 ± 1.7
Pre-pregnancy blood pressure (mmHg)	124 (±26)/64 (±10) (*n* = 9)
Post-pregnancy blood pressure (mmHg)	123 (±13)/68 (±9) (*n* = 9)
Body Mass Index (kg/m^2^)	24.7 ± 5.0
Diabetes	1
Smoking	4

*Table shows demographics of the patients as mean ± standard deviation unless otherwise stated*.

*TA, Truncus Arteriosus; TAP, transannular patch; PVR, pulmonary valve replacement; DORV, double-outlet right ventricle; TOF, Tetralogy of Fallot; AVR, aortic valve replacement*.

### Structural Changes

Four patients (31%) did not present a change in aortic dimensions. The other patients showed median aortic growth of 4 mm (2–7 mm), representing a significant growth compared to pre pregnancy values (*p* = 0.01) based on cross-sectional images (Table 4). Overall, ultrasound measurements confirmed a significant change in aortic dimensions during pregnancy (Table 5).

**Table 4:** Aorta diameters before and after pregnancy.

**CHD**	**Pre_SV** **(mm)**	**Pre_STJ** **(mm)**	**Pre_mid** **(mm)**	**Post_SV** **(mm)**	**Post_STJ** **(mm)**	**Post_mid** **(mm)**
ToF	29	23	27	27	23	29
TA	28	19	25	35	26	30
d-TGA	24	23	21	27	24	26.5
*P*-value (pre_post)				*p* = 0.1	*p* < 0.01	*p* = 0.2

*CHD, congenital heart defect; SV, sinus of Valsalva; STJ, sinotubular junction; TA, truncus arteriosus; d-TGA, transposition of the great arteries*.

**Table 5:** Ultrasound aorta diameters before, during, and after pregnancy.

**CHD**	**Pre_SV(mm)**	**During_SV (mm)**	**Post_SV (mm)**
Arterial switch	28.5	34.5	31
TOF	30	35	33
Atrial switch	26.5	31.5	27
TA	33	38	35
Total (mean)	29.25	34.75	32
*P*-value^*^		0.01	

*CHD, congenital heart defect; SV, sinus of Valsalva; TA, truncus arteriosus; d-TGA, transposition of the great arteries*.

* *P-value represents the difference of the aorta dimeter between before and during pregnancy*.

### Physiological Parameters

**Figure 1:**
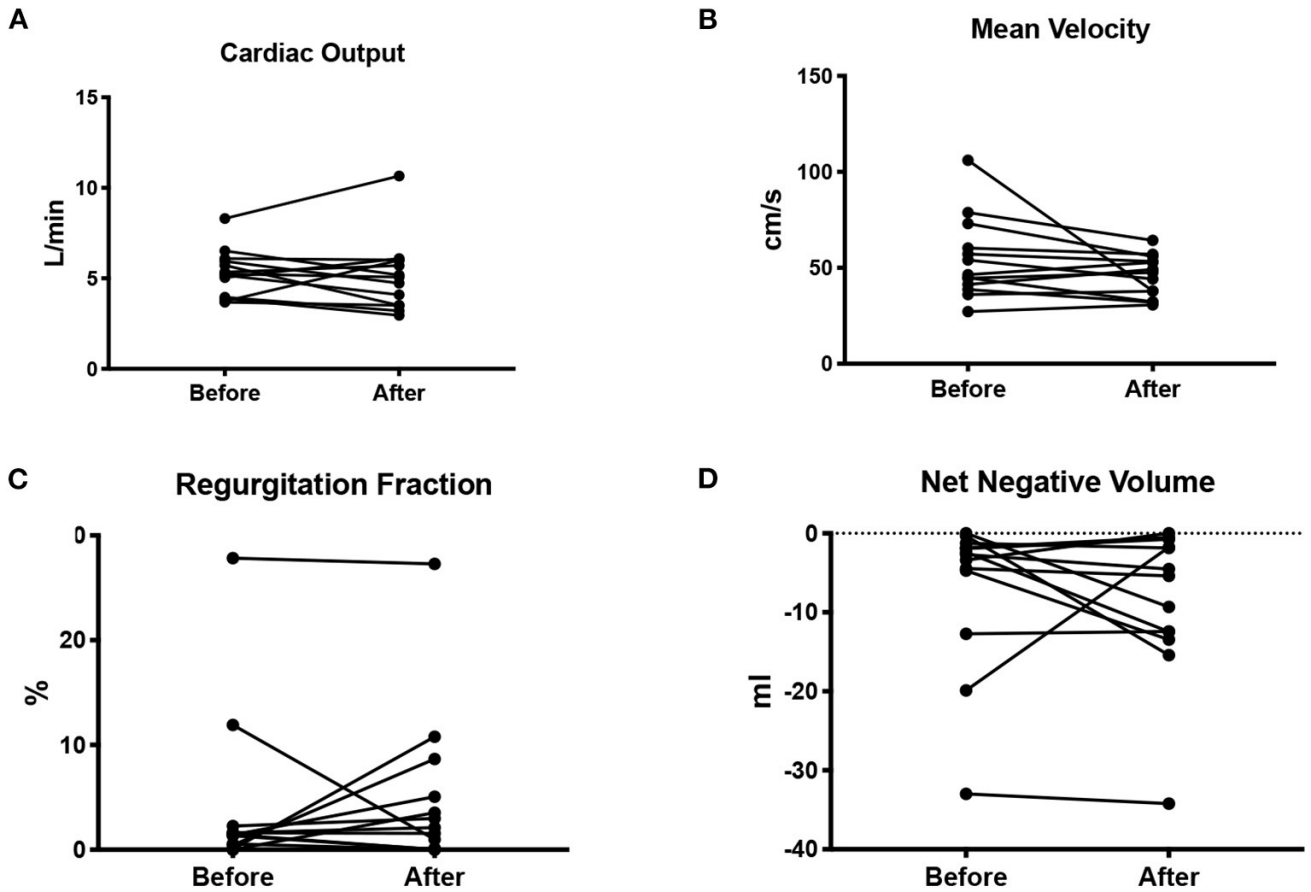
Cardiac output in L/min **(A)**, mean blood flow velocity in cm/s **(B)**, regurgitation fraction as a percentage of the cardiac output **(C)**, and net negative blood volume **(D)** measured before and after pregnancy.

There was no difference in the cardiac output before or after pregnancy (5 ± 1 vs. 4.8 ± 1.5 L/min; *p* = 0.71). There was also no difference in SV/ESV before or after pregnancy (1.7 ± 0.5 vs. 1.9 ± 0.7; *p* = 0.53). Mean velocity across the cardiac cycle (*p* = 0.13), regurgitation fraction (*p* = 0.64), and net negative volume (*p* = 0.38) also did not change after pregnancy. None of the patients presented clinical signs of aortic stenosis. Results are shown in Figure 1.

### Wave Speed

**Figure 2:**
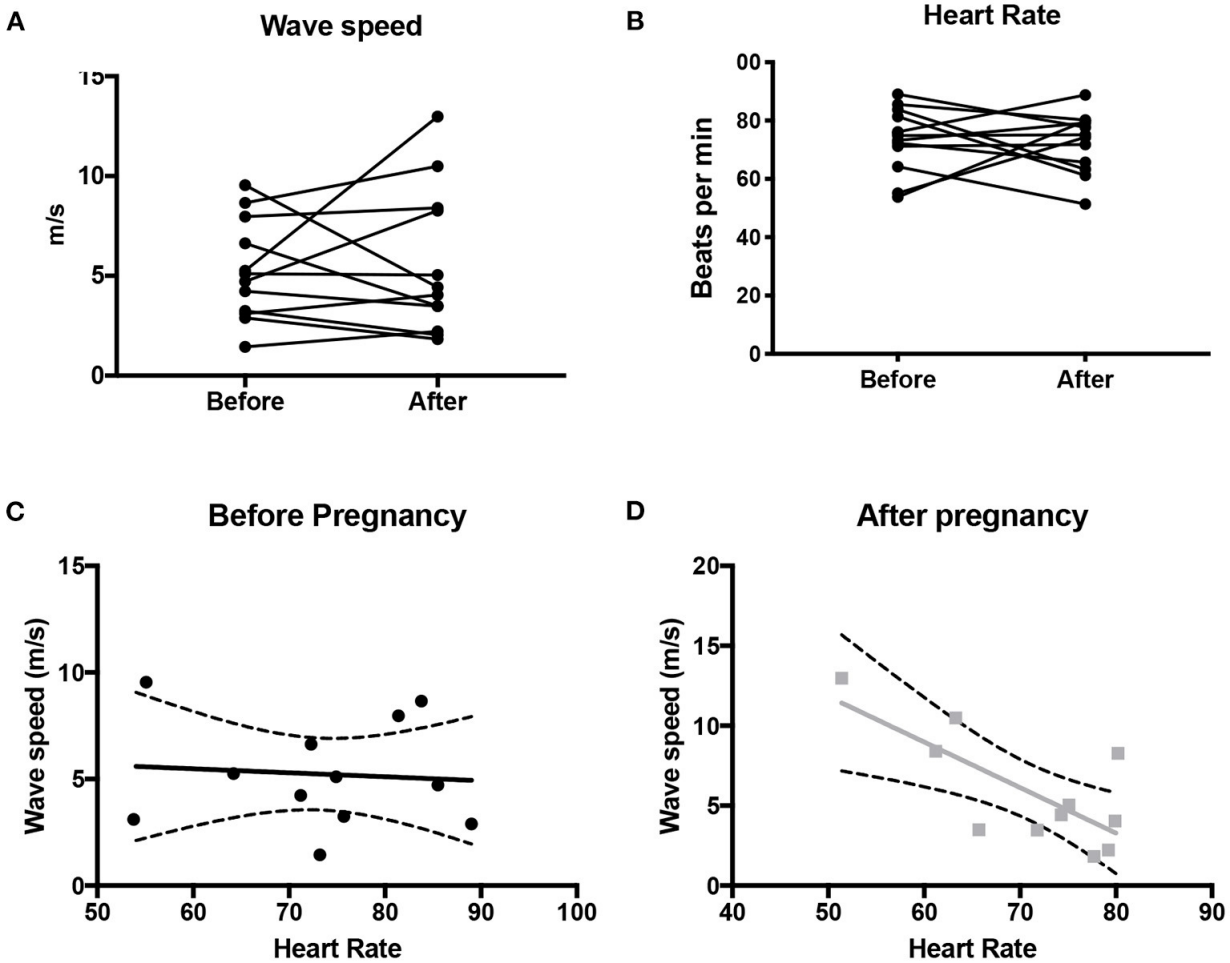
Wave speed **(A)** and heart rate **(B)** in the ascending aorta before and after pregnancy. Also shown is the correlation (+/- 95% CI) between wave speed and heart rate before pregnancy **(C)** and after pregnancy **(D)**.

**Figure 3:**
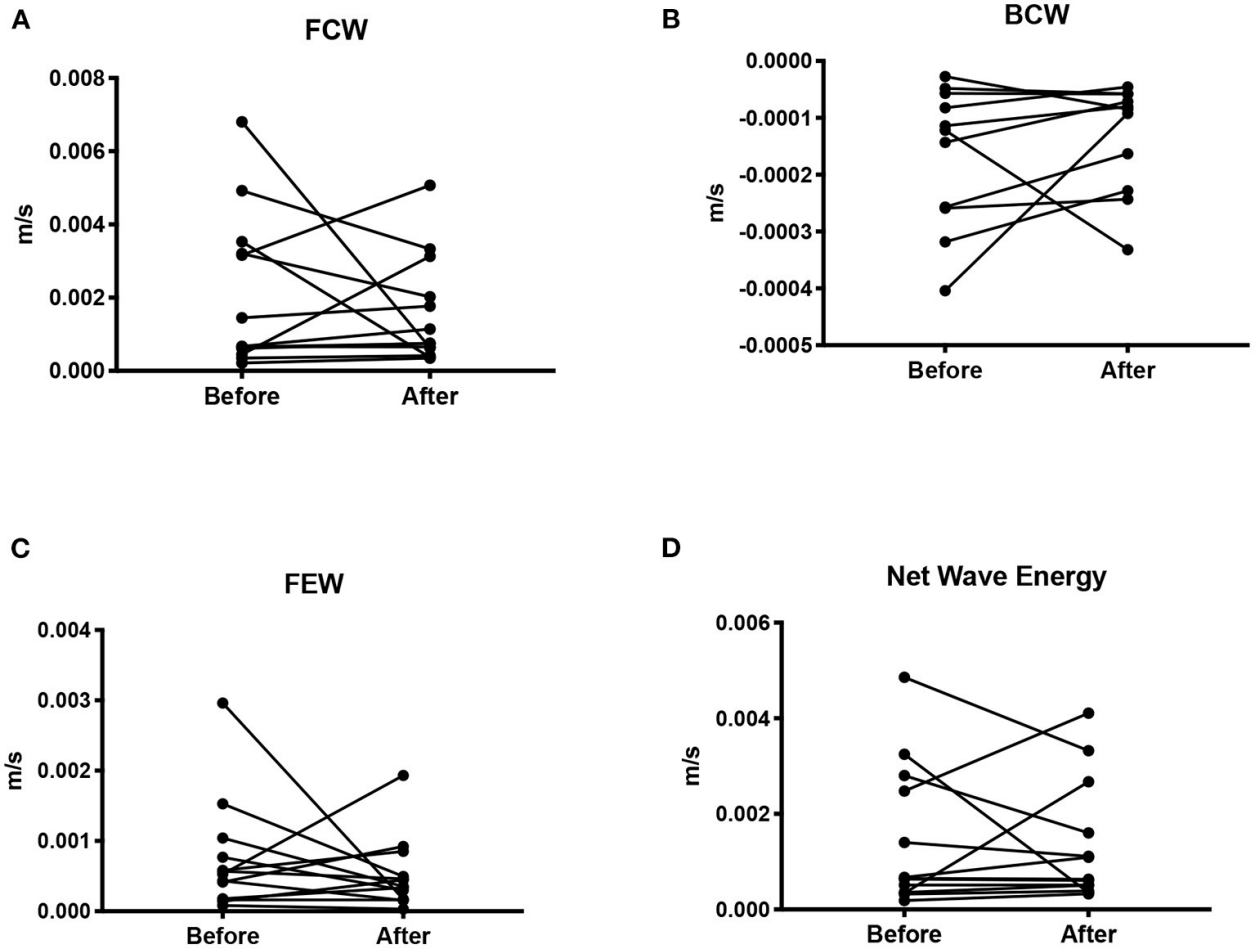
Wave intensity shown as the mean wave energy (AUC) for the forward compression wave **(A)**, backward compression wave **(B)**, forward expansion wave **(C)**, and net wave energy **(D)**.

There was no difference in wave speed before and after pregnancy (*p* = 0.7) (Figure 2). A significant negative correlation was seen between wave speed and heart rate (*p* = 0.01, *r* = −0.73), suggesting that increased stiffness was associated with lower heart rate (Figure 3). This was not observed before pregnancy (*p* = 0.8, *r* = −0.08). Wave speed results are shown in Figure 2.

There was no correlation between cardiac output and wave speed before (*p* = 0.16, *r* = 0.43) or after pregnancy (*p* = 0.057, *r* = −0.56). Similarly, there was no correlation between wave speed and number of pregnancies (pre: *r* = 0.0083, *p* = 0.98; post: *r* = −0.17, *p* = 0.60), pre-pregnancy systolic blood pressure (pre: *r* = −0.11, *p* = 0.79, *n* = 8), or diastolic blood pressure (pre: *r* = −0.42, *p* = 0.30; post *r* = −0.02, *p* = 0.94, *n* = 8). However, a significant correlation was observed between wave speed and systolic pressure post-pregnancy (post *r* = 0.73, *p* = 0.006).

### Wave Intensity

There was no difference in either the FCW (*p* = 0.45), or the FEW (*p* = 0.62) before and after pregnancy. Nor was there a difference in the BCW (*p* = 0.24, *n* = 11) or net WI (*p* = 0.79, *n* = 13) before and after pregnancy.

There was no correlation between FCW, BCW, net *dI* and age, number of pregnancies, SV/ESV, or blood pressure before or after pregnancy. Nor was there a correlation between FEW and age, number of pregnancies or blood pressure before pregnancy. FEW showed a positive correlation between the number of pregnancies (*r* = 0.68, *p* = 0.01).

## Discussion

Patients with congenital heart disease including CTA may present with dilatation of the thoracic aorta during their lifetime. Histological abnormalities of the aortic wall have been identified in patients with ToF and d-TGA as responsible for the early presentation of aorta dilation despite early surgical repair (10, 12, 14, 15).

Tobias et al. have shown that a CTA diagnosis and the presence of aortic regurgitation were independent predictors of an increased aortic sinus diameter (8). Moreover, they add that the diagnosis of a conotruncal defect and age are independent predictors of ascending aorta distensibility. Some independent variables are also known to influence this outcome, such as age, hypertension, smoking, diabetes, and additive pregnancies. These variables are thought to lead to changes in the structure of the aortic media, leaving the aortic wall more vulnerable to dilation and eventually rupture and dissection (10).

There is an association between pregnancy and arterial aneurysm and dissections (9) in females without underlying aortopathy. This risk is secondary to a combination of the hemodynamic stress and hormonal changes imposed during pregnancy on the aortic wall. Nolte et al. reported histological changes in the aortic wall in two women without underlying aortopathy, strongly suggesting that these findings were associated with pregnancy stress plus hormonal changes. It is possible that, after repeated pregnancies, arterial walls might become abnormal because of a combination of hemodynamic stresses and hormonal changes, potentially enhancing the development of pathological changes in the arterial wall. Pregnancy is accompanied by fragmentation of elastic fibers, a decrease in mucopolysaccharides, and an increase in smooth muscle cells in the aortic wall (10). To what extent these changes normalize after delivery is unknown. Since more women with CTAs are reaching childbearing age, it is sensible to investigate if these changes persist temporarily or permanently after pregnancy or even if additive pregnancies enhance the progression of further histological changes.

Persistence of histological changes after pregnancy in CTA patients will lead to increased stiffness and reduced aortic distensibility (10). Stiffness of the aorta will have a negative impact on the ventricular function due to increased afterload, resulting in unfavorable ventriculo-arterial coupling. Recent evidence has suggested that patients with D-TGA and hypoplastic left heart syndrome (HLHS) have unfavorable ventricular-vascular coupling in light of reduced aortic distensibility (11, 12). Moreover, another study, which compared aorta distensibility between arterial and atrial switches, has shown that both groups had reduced distensibility, but the arterial switch cohort presented stiffer aortas. In addition, both had compromised ventricular-arterial coupling based on WIA, likely a result of increased impedance caused by the stiffer ascending aorta (17).

Our study has analyzed aortic distensibility and ventriculo-arterial efficiency in patients with CTA before and after pregnancy, aiming to evaluate whether pregnancy physiology modifies aortic elastic properties and, consequently, mechanical function of the ventricle. No significant changes of aortic distensibility or ventricular-vascular coupling were found. Nonetheless, a significant increase in aortic diameter was seen during pregnancy, based on ultrasound measurements, which persisted 2.3 years post-partum. This is similar to recent evidence that showed that pregnant women with CTA presented with a significant increase in aorta diameter during pregnancy, based on echocardiographic measurements, without reversion to a baseline diameter after 6 months of follow-up (18).

Wave speed has shown a positive correlation with HR, suggesting that higher wave speed presents with lower heart rate in this population. We can suggest that, in CTA patients, heart rate tends to decrease over time in the context of sinus node dysfunction, mainly in patients with congenitally corrected (CCTGA) and D-TGA who have undergone a Mustard or Senning operation. Four out of 13 patients decreased more than 10 beats per minute after pregnancy, of which three were D-TGA patients, in whom increased aortic stiffness over time has also been reported (18). Nonetheless, it is known that heart rate can be a confounding variable that varies depending on systolic blood pressure measurements (19, 20). Further investigation is needed in order to fully explore this observation in a larger sample.

Furthermore, wave speed was found to be associated with systolic blood pressure after pregnancy in this cohort. An increment in systolic blood pressure could be associated with the aging process, albeit over a small number of years and other risk factors associated such as diabetes, smoking, or BMI >25 kg/m^2^. We observed that patients with increased systolic blood pressure post-pregnancy were overweight (four out of 13 had a BMI ≥ 25 kg/m^2^ post-pregnancy), two were smokers, and another was diabetic.

Another interesting finding was the significant positive correlation between FEW and number of pregnancies. While again limited by the small sample size, this could be clinically interesting given that FEW has been previously associated with the diastolic time constant tau (16). This would suggest that repetitive pregnancies may improve ventricular compliance, thus reflecting diastolic function. There is a possible explanation to address this finding: preload increases during pregnancy that lead to an increment in the stretching mechanism of the myocardium that regresses after 6–8 weeks post-delivery (18). After repetitive pregnancies, we can assume that ventricular compliance reaches the maximum point of stretching based on the Frank Starling mechanism, leading to more compliant ventricles. This remains speculative at this stage, as observed in a small number of cases, and should be explored in a larger sample allowing for sufficient power for subgroup analysis.

Indeed, the major limitation of this study is the small available sample, due in part to the number of patients with CTA undergoing pregnancy as well as the availability of the required imaging data. If a larger sample were available, it would be interesting to explore some of the associations mentioned here, particularly the possible association between wave energy (both FCW and FEW) and SV/ESV as a different CMR-derived indicator of ventriculo-vascular coupling.

Larger samples would likely require multicenter collaborations, thus allowing the exploration of the effect of multiple pregnancy or subtle differences between different diagnoses within CTA. Nevertheless, the study demonstrates the feasibility of performing the analysis in this patient population and the kind of physiological insight that could be gathered with regards to functional changes post pregnancy and the overall impact on ventriculo-arterial coupling.

From a methodological standpoint, it is worth remembering that several methods can be employed for the calculation of wave propagation, and even with a non-invasive approach, different techniques could be employed, with respective advantages and pitfalls (21). While an in-depth comparison and discussion of different techniques are beyond the scope of this article, it is worth considering that research is addressing the consistency and robustness of regional and local CMR-derived indices according to the theoretical model (Bramwell-Hill), demonstrating their relevance (22). Interestingly, WIA has been recently performed by combining standard CMR flow-velocity and non-invasive central blood pressure waveforms (23), but our study was purposely based solely on clinically available CMR data. Furthermore, measures of net wave intensity do not require knowledge of wave speed and thus are not influenced by the wave speed measurements.

## Conclusion

Aortic distensibility did not change after pregnancy in CTA patients, but an increase in aortic diameter was observed. Serial measurement of the aortic root during and after pregnancy is clearly important. Wave intensity analysis is feasible in this population and could provide physiological insights in larger cohorts.

## Data Availability Statement

The original contributions presented in the study are included in the article/supplementary materials, further inquiries can be directed to the corresponding author/s.

## Ethics Statement

This was a retrospective feasibility study performed at a single NHS site. Therefore, no approval from NHS UK research and ethics committee was sought in line with the Health Regulatory Authority guidelines.

## Author Contributions

MO: contributed with the idea, writing, analysis of the wave intensity analysis results, and the clinical significance. SN: did the analysis of the data and the wave intensity analysis. MC: supervised the study and writing. SC: lead the pregnancy input and helped in the writing process. GB: coordinated the study and writing and analysis of the data. All authors contributed to the article and approved the submitted version.

## Conflict of Interest

The authors declare that the research was conducted in the absence of any commercial or financial relationships that could be construed as a potential conflict of interest.
